# Reproducible brain PET data analysis: easier said than done

**DOI:** 10.3389/fninf.2024.1420315

**Published:** 2024-09-30

**Authors:** Maryam Naseri, Sreekrishna Ramakrishnapillai, Owen T. Carmichael

**Affiliations:** ^1^Department of Physics and Astronomy, Louisiana State University, Baton Rouge, LA, United States; ^2^Biomedical Imaging Center, Pennington Biomedical Research Center, Baton Rouge, LA, United States; ^3^Department of Radiology & Biomedical Imaging, Yale School of Medicine, New Haven, CN, United States; ^4^Department of Neurological Surgery, University of Pittsburgh, Pittsburgh, PA, United States

**Keywords:** reproducible science, reproducibility crisis, brain PET, positron emission tomography, scientific workflows, pre-registration, community standards

## Abstract

While a great deal of recent effort has focused on addressing a perceived reproducibility crisis within brain structural magnetic resonance imaging (MRI) and functional MRI research communities, this article argues that brain positron emission tomography (PET) research stands on even more fragile ground, lagging behind efforts to address MRI reproducibility. We begin by examining the current landscape of factors that contribute to reproducible neuroimaging data analysis, including scientific standards, analytic plan pre-registration, data and code sharing, containerized workflows, and standardized processing pipelines. We then focus on disparities in the current status of these factors between brain MRI and brain PET. To demonstrate the positive impact that further developing such reproducibility factors would have on brain PET research, we present a case study that illustrates the many challenges faced by one laboratory that attempted to reproduce a community-standard brain PET processing pipeline. We identified key areas in which the brain PET community could enhance reproducibility, including stricter reporting policies among PET dedicated journals, data repositories, containerized analysis tools, and standardized processing pipelines. Other solutions such as mandatory pre-registration, data sharing, code availability as a condition of grant funding, and online forums and standardized reporting templates, are also discussed. Bolstering these reproducibility factors within the brain PET research community has the potential to unlock the full potential of brain PET research, propelling it toward a higher-impact future.

## 1 Introduction

Science thrives on incremental progress, where the creation and organization of knowledge is driven by a cycle of developing theories and testing predictions made by those theories. When tests of a prediction have the same result repeatedly, confidence in the test result grows, especially when the tests are run by different scientists operating independently. Several terms have been introduced to describe different variants of this test repetition concept: a test result is considered “reproducible” when the same raw measurements and analytic methods are used by a different scientist; “replicable” when new measurements are recorded but the same methods are used as in a previous test; “robust” when the same raw measurements but different analytic methods are used, and “generalizable” when new measurements are recorded and new methods are utilized (Nosek et al., 2022; Poldrack et al., 2019b). In recent years, low levels of reproducibility have been reported in a growing number of scientific disciplines, culminating in a so-called “reproducibility crisis” (Ioannidis, 2005; Munafò et al., 2017). Neuroscience, and specifically *in-vivo* human neuroimaging, is no exception to this trend. Despite the increasing volume of neuroimaging publications, significant doubts about their accuracy and generalizability have emerged (Poldrack et al., 2017; Klapwijk et al., 2021; Botvinik-Nezer et al., 2020). Three studies (Carp, 2012; Guo et al., 2014; Poldrack et al., 2017) of methodology reporting in functional Magnetic Resonance Imaging (fMRI) papers found that researchers often omit important details like interpolation methods, smoothness estimates, and multiple comparison correction techniques. Neuroimaging researchers have also suggested that low statistical power (Müller et al., 2017; Button et al., 2013; Hong et al., 2019), hypothesizing after the results are known (Klapwijk et al., 2021), and publication bias (David et al., 2013) have contributed to reproducibility limitations (Bishop, 2019; Munafò et al., 2017). These shortcomings have highlighted the need to implement a set of specific factors that will encourage robust and reproducible findings in neuroimaging data analysis. The next section will explore these reproducibility factors and compare their current status between fMRI and brain PET fields.

## 2 Reproducibility factors relevant to brain PET

In neuroimaging research communities, a set of reproducibility factors have been proposed as a means to promote explicit documentation of methods, availability of research materials, pre-registration of hypotheses and analyses, and standardized analysis pipelines, with an end goal of enabling easier replication of findings (Jadavji et al., 2023). In subsequent sections, we define these factors, explore their current status within neuroimaging in general, and then describe how prevalent they are in both fMRI and brain PET communities.

### 2.1 Standards for writing and publishing scientific studies

Scientific papers serve as the primary means for sharing research findings. Accurate and complete descriptions of methods and data versioning allow fellow researchers to understand exact procedures, exact data/software versions and underlying rationale, facilitating error detection and incremental improvement in study design or data analysis (Munafò et al., 2017; Gorgolewski and Poldrack, 2016; Aguinis et al., 2018). The Organization for Human Brain Mapping Committee on Best Practices in Data Analysis and Sharing (COBIDAS) actively advocates for journals to abide by best practices in the reporting of neuroimaging methods and data (Poldrack et al., 2017). Specialized committees have developed reporting standards that are specific to structural MRI and fMRI (Nichols et al., 2017), Magnetoencephalography (MEG) (Pernet et al., 2020), Electroencephalogram (EEG) (Styles et al., 2021), and Positron Emission Tomography (PET) (Knudsen et al., 2020). Web-based apps like pyBIDS, bids-matlab, and fMRIPrep can help authors adhere to these guidelines by automatically generating reports and providing method templates (Niso et al., 2022a). Additionally, automatic tools like *statcheck* can help identify inconsistencies in statistical results reported in research papers by extracting the test statistic and degrees of freedom and recalculating *p*-values. This helps ensure the statistical results are accurate (Nuijten and Polanin, 2020; Epskamp and Nuijten, 2016).

#### 2.1.1 Current state of fMRI

A recent review of 160 fMRI publications found that most of them had adopted reporting practices recommended in 2016 by COBIDAS, including clear descriptions of study design (85%), motion correction (77%), and registration to a standard space (80%) (Acar et al., 2023).

#### 2.1.2 Current state of PET

Despite initial steps toward establishing reporting standards, including a panel discussion at the 2016 NeuroReceptor Mapping conference and a subsequent consensus paper (Knudsen et al., 2020), progress has been limited. Although a PET standardized reporting checklist project (eCOBIDS PET) was initiated, as of 2024, it remains incomplete and still under development (COBIDAS Contributors, 2020).

### 2.2 Pre-registration

Pre-registration entails writing a study plan that includes information about planned data acquisition, inclusion and exclusion criteria, analytic methods, and depositing that study plan in a third-party repository prior to data acquisition (Poldrack et al., 2017). The goal is to ensure that after the data is collected, publications containing the data use the pre-registered study plan to prove which of their analyses were *a priori* hypothesis driven as opposed to exploratory and thus susceptible to analytic biases (Gorgolewski and Poldrack, 2016). There are three main platforms currently used by neuroimaging studies for pre-registration (OSF, 2011; NIH, 1997; Wharton-Upenn, 2017) and the Center for Open Science provides detailed forms and guidelines to support pre-registration (COS, 2013). An alternative to the pre-registered study plan is the registered report (RRs), which allows authors to submit methodology text for peer review and conditional acceptance prior to data analysis. Initial studies suggest that RRs in neuroscience outperform traditional papers in methodological rigor, analysis quality, and overall paper quality (Soderberg et al., 2021). Leading neuroimaging journals including Neuroimage have recently incorporated RRs as a new article type (NEUROIMAGE, 2024).

#### 2.2.1 Current state of fMRI

A 2022 survey of 283 fMRI researchers worldwide revealed a mixed picture regarding pre-registration. Some 57.6% had pre-registered an fMRI study, and 14.1% had written an RR. In addition, out of those who have pre-registered their studies, only 55% expressed willingness to pre-register their next study, and 26% were hesitant to do so (Paret et al., 2022).

#### 2.2.2 Current state of PET

The brain PET community does not appear to have embraced pre-registration yet. Of the 32 neuroimaging studies that were pre-registered on the OSF website between Aug 2019 and Jan 2022, all involved structural MRI and/or fMRI, and none involved PET (OSF, 2011). In addition, the main platforms for pre-registration include study plan templates tailored for fMRI, but not for PET.

### 2.3 Data sharing

Sharing raw and/or processed data empowers researchers to validate published findings, explore new methodologies, and conduct meta-analyses, boosting both transparency and research potential. Pooled data from multiple studies enables investigation of biases and heterogeneity, leading to more reliable findings. Increasingly, journals and funding agencies mandate data sharing, promoting scientific advancement. Numerous publicly available neuroimaging datasets (ADNI, 2004; HCP, 2011; Openneuro, 2022; Marcus et al., 2007) underscore the value of these resources for researchers. In this context, the Brain Imaging Data Structure (BIDS) has established a consensus on organizing and sharing MRI and fMRI data (Gorgolewski et al., 2016). They later introduced extensions of BIDS to include MEG (Niso et al., 2018), EEG (Pernet et al., 2019), and PET (Norgaard et al., 2022).

#### 2.3.1 Current state of fMRI

In the current state of fMRI, data sharing practices vary, with 66% of participants sharing raw data but only 54% intending to share data from their next paper, citing barriers such as consent forms and institutional review boards (Paret et al., 2022).

#### 2.3.2 Current state of PET

Data sharing in PET is limited. OpenNeuro (Markiewicz et al., 2021) hosts 92 fMRI datasets compared to just 9 for PET. Even access to existing PET data faces hurdles: PET scans with newer radiotracers often face stricter limitations on data sharing, and some institutions limit sharing to only those researchers that collaborate with their investigators. While repositories like ADNI (Jagust et al., 2015) and A4 (Insel et al., 2020) host a large number of brain PET scans, there is still a relative lack of large datasets featuring recently-developed radiotracers.

### 2.4 Codes, containers, and the cloud

Traditional publications necessarily provide abbreviated descriptions of computational methods applied to neuroimaging data, due to space constraints. Therefore, the most accurate source of information about every detail of computational procedures is the source code of the computer programs themselves (Niso et al., 2022a; White et al., 2022). Version control system repositories such as the GitHub and Brainlife offer platforms for open code sharing and preserving snapshots of specific versions (Kubilius, 2014), thus encouraging reproducibility by enabling differing labs to implement precisely the same computational steps. Beyond code that governs individual computational steps, the complexity of neuroimaging analysis workflows that assemble sequences of such steps presents challenges for reproducibility (Paninski and Cunningham, 2018). These workflows rely on intricate software dependencies (Merkel, 2014), system-level resources (Abe et al., 2022), and specialized packages (Poldrack et al., 2019a) which can lead to inconsistencies across computing environments and divergent results (Renton et al., 2022; Halchenko and Hanke, 2012; Niso et al., 2022a). Software containers such as Docker and Singularity have emerged as effective tools for capturing entire software stacks, ensuring reproducibility across different platforms (Kurtzer et al., 2017). Additionally, BIDS Apps enhance reproducibility by packaging existing neuroimaging pipelines in containers, resolving installation issues, and improving analysis reproducibility (Gorgolewski et al., 2017).

#### 2.4.1 Current state of fMRI

An fMRI survey (Paret et al., 2022) showed that out of 183 participants, 66% have used containerized BIDS Apps dominated by tools like fMRIPrep (44%) and MRIQC (23%) highlighting a thriving ecosystem for code and software sharing. fMRIPrep generates visual reports to assess result quality and aids researchers in understanding each workflow step. The reports include comprehensive text descriptions of major pipeline steps, including exact software versions and citations.

#### 2.4.2 Current state of PET

In contrast to the extensive support available for fMRI, there is a lack of dedicated PET software in containerized BIDS Apps and limited support for PET code sharing in platforms like Brainlife.

### 2.5 Optimizing and standardizing workflows

Quantifying brain activity requires complex analysis pipelines, and researchers have significant flexibility to customize each pipeline step. This flexibility, however, can lead to vastly different results from the same data, especially in fMRI studies (Loring et al., 2002; Eklund et al., 2016; Botvinik-Nezer et al., 2020). The choice of analytical methods also plays a crucial role in the variability of findings across neuroimaging techniques. For example, differences in approaches such as intensity-based versus feature-based coregistration (Li et al., 2024), gradient descent-based versus trust region-based optimization methods (Zhao and Xie, 2016), or PET template-based versus MRI-based image processing (Kuhn et al., 2014) can each act as a distinct reproducibility factor, independent of the software package used to implement these methods. Studies in PET (Greve et al., 2016; Mukherjee et al., 2016; Samper-González et al., 2018) also demonstrate how variations in processing workflows can significantly impact results. This variability makes it difficult to distinguish between true effects and biases introduced by analytic choices.

To address this, the neuroimaging community has proposed solutions such as “multiverse analysis” where data is processed through multiple pipelines and all results are combined to identify convergent findings (Dafflon et al., 2022), “pipeline optimization tools” that automatically find the best suited pipeline for a given problem domain to maximize reproducibility (Churchill et al., 2012), and “gold standard pipelines” that become the expected workflow for a specific research task (Niso et al., 2022a). These strategies aim to limit a researcher’s ability to bias the outcome of a study through careful pipeline adjustment.

#### 2.5.1 Current state of fMRI

FMRI reproducibility has been boosted by established pipelines like fMRIPrep (Esteban et al., 2019) and initiatives like HALFpipe (Waller et al., 2022) which promote consistent analysis across studies. However, researchers still navigate complex analysis choices. Multiverse analysis studies (Demidenko et al., 2024; Kristanto et al., 2024) explore the impact that these choices have on results.

#### 2.5.2 Current state of PET

Achieving standardization of analysis pipelines in PET lags behind fMRI. In one study, 14 international groups were asked to analyze the same simulated brain PET dataset using their own methods. Despite controlling for several preprocessing steps using the simulated data, the results were consistent but not identical across the groups. This highlights the significant impact of analytical and statistical choices on PET neuroimaging findings (Veronese et al., 2021). A recent review (Niso et al., 2022a) highlights the limited number of dedicated PET pipelines compared to those for MRI/fMRI. To our knowledge, the development of gold standard pipelines and multiverse analysis is extremely limited in brain PET.

### 2.6 Summary

In summary, while pressure is growing from funding bodies, research institutions, and publishers to implement reproducibility factors within neuroscience (Frank et al., 2017; Niso et al., 2022a), fMRI is currently leading the way and brain PET is struggling to catch up. The following section aims to demonstrate the real-world impact of brain PET’s position as a straggler in reproducibility. We describe how difficult it was to implement a prominent PET analysis pipeline based on available descriptions, to demonstrate that the relative lack of brain PET reproducibility infrastructure has real effects on the conduct of science.

## 3 Case study

**Figure 1:**
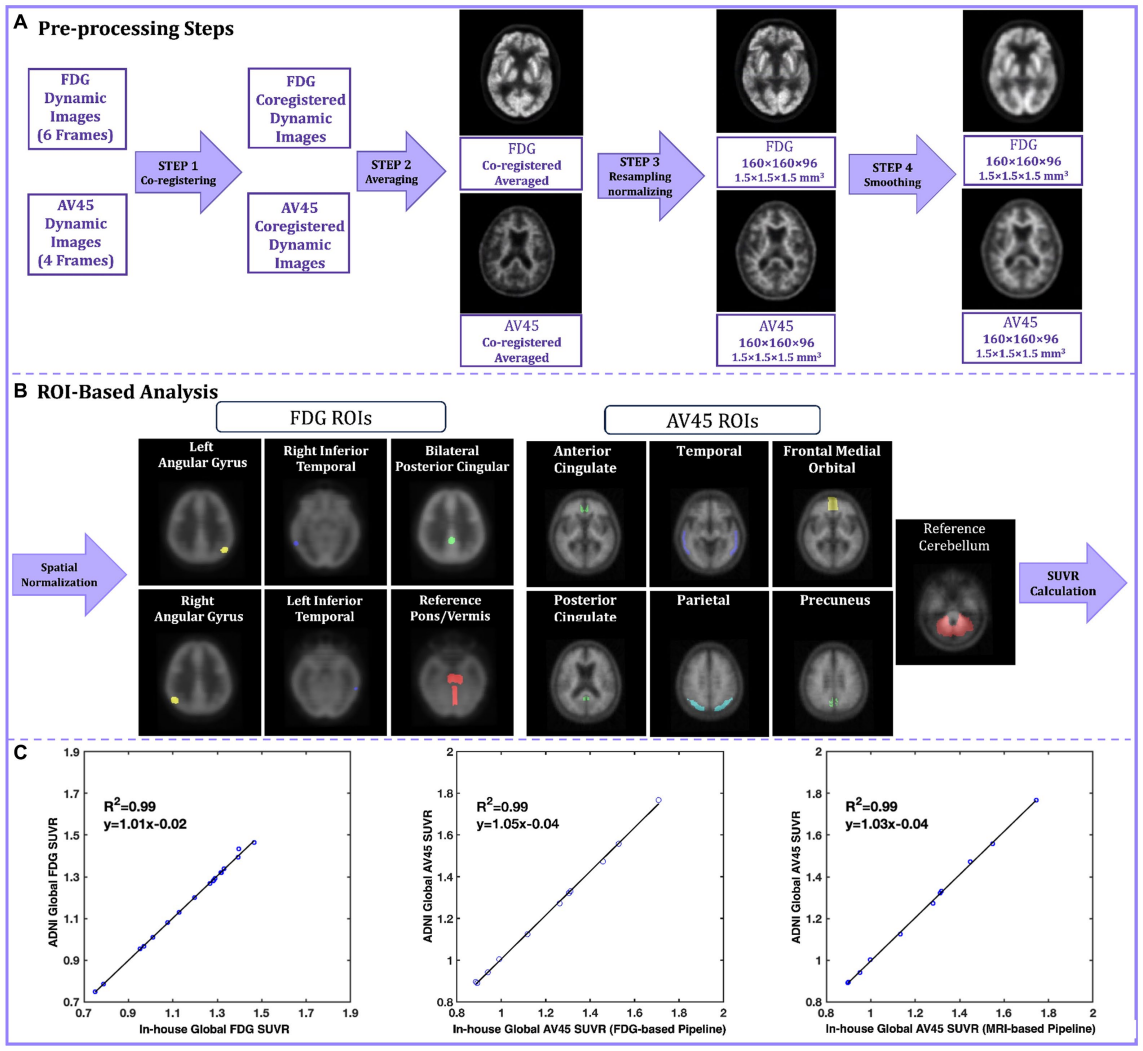
**(A)** The ADNI preprocessing pipeline steps for FDG and AV45. In step 1 original FDG and AV45 frames are coregistered to the reference frame, in step 2 coregistered FDG and AV45 frames are averaged, in step 3 the averaged image is re-grided into 160 × 160 × 96 grid with voxel size of 1.5 mm^3^ to create the baseline FDG and AV45 scans and further intensity normalized, in step 4 the resampled images are smooth using a non-isotropic gaussian filter; **(B)** The ADNI ROI-based analysis pipelines for FDG and AV45; The fully preprocessed (output of step 4) images are spatially normalized into the MNI standard space and pre-defined ROI are applied in respect to the tracer, finally a global SUVR is calculated from the average of the individual ROI SUVRs relative to a reference region of choice; **(C)** The SUVR linear regression plots for the validation dataset. The Y-axis represents ADNI’s calculated global SUVR as the gold standard, while the X-axis represents our in-house calculated global SUVRs for FDG (left), AV45 FDG-based (center), and AV45 MRI-based (right) pipelines. ADNI, Alzheimer’s Disease Neuroimaging Initiative; florbetapir, AV45; ROI, Region of interest; MNI, Montreal Neurological Institute; SUVR, standard uptake value ratio.

To give an example of the real-world experience of PET image processing reproducibility, we tried to reproduce the processing pipeline of a large public dataset from the ADNI study. ADNI offers pre-processed PET scans for 18F-FDG and 18F-Florbetapir (AV45) with incremental levels of processing. Figure 1A illustrates the pre-processing steps for both FDG and AV45 PET scans. A detailed listing of acquisition parameters and description of the pre-processing pipeline can be found on (ADNI, 2004). The original scans include six 5 min FDG and four 5 min AV45 dynamic frames. In step 1 of pre-processing, coregistered dynamic frames are generated, using the first frame as the reference, to correct for head motion. Step 2 averages coregistered dynamic frames to reduce noise. Step 3 spatially reorients the averaged image to a standard grid (160 × 160 × 96 voxels, 1.5 mm^3^). The re-gridding corrects for inter-individual differences in brain size and shape while also providing a consistent baseline across individuals for measuring longitudinal change. The FDG scans are re-grided using an FDG PET Talairach atlas template, while the AV45 scans are coregistered to either the baseline FDG scan (FDG-Based) or the MRI scan of the participant when FDG is not available (MRI-based). Re-gridded, coregistered, dynamic images are then generated by co-registering the original frames to this baseline scan, followed by averaging. Then intensity normalization is applied. The final preprocessing step is to smooth images to ease cross-scanner comparability (Jagust et al., 2015). In the ADNI pipeline, NeuroStat is the software that handles coregistration, re-gridding, and frame co-registration (NeuroStat, 2000), while ADNI’s in-house software performs smoothing, averaging, and intensity normalization. In our replication of the ADNI pipeline, we employed SPM12 (MATLAB, 2020b) for smoothing and averaging. We skipped the initial intensity normalization in step 3 because this simply put voxel values on a uniform scale, and the subsequent analyses involve their own normalization. ADNI uses a separate in-house intensity normalization for amyloid scans, so we focused on replicating the core pre-processing steps and this omission did not affect the final results.

Quantification of fully preprocessed scans involved region of interest (ROI) based analyses using ADNI’s method for FDG and AV45 (Figure 1B). Both scans were spatially normalized to MNI space in SPM12 using its default FDG template (Della Rosa et al., 2014) and a specialized florbetapir template (Joshi et al., 2015). Standard uptake value ratios (SUVRs) were calculated for specific ROIs. The FDG ROIs are comprised of the right/left angular gyrus, right/left inferior temporal gyrus, and bilateral posterior cingulate in reference to pons/cerebellar vermis region (Landau et al., 2011). The AV45 ROIs are comprised of the medial orbital frontal, temporal, parietal, anterior cingulate, and posterior cingulate cortices as well as the precuneus, with the entire cerebellum as a reference region (Joshi et al., 2015). Finally, a global SUVR is computed from the average of the individual ROI SUVRs. The dataset we validated included 11 subjects with diagnoses ranging from cognitively normal (*n* = 6) to mild cognitive impairment (*n* = 5). All participants had FDG, AV45, and T1-weighted MRI scans, and for all participants, both their FDG and AV45 PET scans were included in this replication study.

For FDG PET pipeline validation, the SUVRs provided publicly by ADNI were used as the gold standard and compared against our in-house calculated SUVRs. For AV45 PET validation, we could not use the ADNI SUVRs because for some part of the ROI-based analysis, they had used SPM2, an outdated software version that is no longer widely available and easy to deploy. Instead, we downloaded the fully pre-processed AV45 PET scans from ADNI and performed ROI-based analysis. These calculated SUVRs on the fully pre-processed ADNI AV45 images were used as the gold standard against our in-house calculations using the unprocessed images of the same AV45 scans. Gold-standard SUVRs were compared to in-house calculated SUVRs using R-squared values from linear regression, and intra-class correlation coefficients (ICCs). Our results demonstrated strong linearity for FDG, AV45 FDG-based, and AV45 MRI-based global SUVRs, with R-squared of 0.99, 0.99 and 0.98, and ICCs of 0.998, 0.996, and 0.998, respectively (Figure 1C).

## 4 Challenges in replicating ADNI PET processing pipeline

While our experimental study ultimately showed strong correlation between in-house and gold standard SUVRs for both tracers, replicating ADNI’s preprocessing and processing pipeline presented significant hurdles. Despite achieving high linearity and ICCs, incomplete documentation and ambiguous implementation details delayed our replication process. These challenges can be categorized into the following areas:

Standards for Writing and Publishing Scientific Studies:

- Lack of specification for intermediate steps, design choices, and software parameters and settings in ADNI documentation.
- Undocumented sub-steps in the ADNI pipeline, such as pre-smoothing frames before coregistration in step 1 and re-gridding AV45 scans using MRI when FDG scans are unavailable in step 3.

Data Sharing:

- Compatibility issues arose with NeuroStat requiring ECAT format files not available from the ADNI website.
- Obtaining undocumented, crucial information necessitated direct contact with the ADNI PET group.

Codes, Containers, and the Cloud:

- Transitioning between different software tools (e.g., NeuroStat and SPM) presented challenges due to varying data format requirements.
- Irreproducibility of ADNI’s normalization approach for AV45 scans due to the utilization of specific ADNI in-house software.
- Inability to use ADNI Avid SUVRs as the gold standard for AV45 pipeline validation due to outdated SPM2 version.

Optimizing and Standardizing Workflows:

- The use of ADNI’s in-house software for normalization made it difficult to replicate their exact approach, highlighting the need for standardized techniques.

## 5 Discussion

Our perspective and case study showed that PET neuroimaging research faces a concerning reproducibility gap compared to fMRI and MRI. While the sheer volume of research using fMRI and MRI contributes to this disparity, other factors are also at play. PET data often presents unique challenges due to lower signal-to-noise ratios, partial volume effects, and specialized reconstruction techniques. Additionally, the diverse clinical and research applications of PET contribute to a more heterogeneous research landscape compared to fMRI. The rapid evolution of PET technology further complicates standardization efforts.

Efforts within the PET community to address these challenges are ongoing. For instance, Greve et al. highlight how variations in partial volume correction methods can significantly impact results, underscoring the need for standardized approaches (Greve et al., 2016). Initiatives like the NeuroReceptor Mapping conference and the consensus paper also aim to develop guidelines and frameworks for PET research, although the field still lacks comprehensive and universally adopted standards (Knudsen et al., 2020). Pfaehler et al. also point out that the lack of publicly available data, heterogeneity in metrics, and insufficient reporting details hinder reproducibility in radiomic studies. They call for standardized preprocessing steps to improve comparability and reproducibility across studies (Pfaehler et al., 2021). A recent review emphasizes that while various organizations have developed harmonization strategies for quantitative PET, international methodology harmonization is still needed to ensure comparability across global clinical studies (Akamatsu et al., 2023).

Compared to PET, EEG and MEG (MEEG) have made strides in standardization, particularly regarding acquisition protocols, reporting, and analysis pipelines (Niso et al., 2022b; Gross et al., 2013). However, all these neuroimaging fields encounter obstacles related to data sharing, consistent analysis methods, and collaborative culture. As highlighted by the LiveMEEG 2020 conference (Niso et al., 2022b), a collaborative mindset is essential for advancing reproducibility in neuroimaging.

The MEEG community has demonstrated the value of shared efforts, emphasizing the importance of resource sharing, knowledge exchange, and joint problem-solving. While PET research has begun to embrace open science principles, there is a need for a more concerted and collaborative approach. The PET community must embrace open science practices like comprehensive documentation, open-source software, detailed pipeline descriptions, and dedicated communication platforms. Implementing stricter standards and data sharing policies across PET journals is crucial for fostering a cultural shift toward open science. The limited standardized reporting checklists, pre-registration, containerized tools, and standardized processing pipelines exacerbates the issue. Initiatives like OASIS and ADNI have made strides in accumulating PET scans, but sharing data from newer radiotracers, such as flortaucipir, is crucial for advancing PET research. Promoting the development of standardized pipelines and tools should be a top priority. Solutions like incentivizing open practices by designating funding for long-term maintenance of code and data repositories, as well as, offering promotions, tenure, and influencing funding decisions based on data reuse can drive progress.

## Data Availability

The original contributions presented in the study are included in the article/Supplementary material, further inquiries can be directed to the corresponding author.
