# Supplementary material for: Reproducible brain PET data analysis: easier said than done
Source: Front Neuroinform. 2024 Sep 30;18:1420315. doi: 10.3389/fninf.2024.1420315 (PMC11472777; doi:10.3389/fninf.2024.1420315)
Supplement: Supplementary file 1 [file Data_Sheet_1.docx]

Preprocessing Pipeline

Step 1: Coregistered dynamic images

Each 18-F FDG scan, has six five-minutes frames and each 18F-florbetapir scan, has four five-minutes frames. The original frames were downloaded in NIFTI format and then converted to Analyze (hdr/img) format using *imcal* function in SPM 12 (MATLAB 2020b) which is a compatible format for Neurostat software. In the preprocessing pipeline, first, all the frames were smoothed with an isotropic [8 8 8] Gaussian filter to get the resolution of 8 mm full width at half maximum (FWHM) using *smooth* function in SPM 12 (MATLAB 2020b). This smoothing was done in order to get more accurate motion vectors since the single frames are very noisy by nature and contains lower number of counts. Therefore, the coregistration was performed on the smoothed images to improve the quality of the coregistration. Afterwards, the transformation vectors were used to co-register all the original frames to the first original frame. All the smoothed frames were co-registered to the first smoothed frame using *mcoreg* function in Neurostat software (MacOSXLion). This function provides a rigid body co-registration with 6 degrees of freedom for translation/rotation. Cost function of 3 was selected (for intramodality coregistration). The motion vectors between frames for the three translation and three rotation parameters were stored and used to co-register all the original frames to the first frame using *coregimg* function in Neurostat. Figure 1S shows an example of this step for an FDG scan. Note that all images in set one is in their native space.

Step 2: Coregistered and Averaged

In this step all the coregistered frames were averaged to form a single image that is less noisy and has increased number of counts. This type of preprocessed image set is generated simply by averaging the 6 five-minute FDG frames and five-minutes AV45 frames of the set 1 using SPM 12, *imcal* function. The output of step 2 is a coregistered-averaged static image which is still in the image native space. See Figure 2S for an example of step 2 for an FDG scan.

**Figure ‎1S.** First preprocessing step. Original FDG frames (sagittal and coronal views) (top left) are smoothed with an 8 mm isotropic filter (top right). All the smoothed images were co-registered to smoothed frame 1(right bottom) and the coregistration parameters (transformation vectors) were used to co-register all the original frames to the original frame 1 (left bottom).

**Figure 2S.** Second preprocessing step. Six five-minutes coregistered original FDG frames (sagittal and coronal views) (left) are averaged to create the coregistered-averaged FDG image (right).

Step 3: Coregistered, Re-grided, and Averaged

The purpose of this step was to create a standardized image matrix by fixing the spatial orientation of each coregistered-averaged PET image so that longitudinal images from different scanners for a single participant could be compared easily. This was done by spatially reorienting the coregistered-averaged PET image axis such that it is parallel to the anterior commissure and posterior commissure (AC-PC) line. The AC-PC line is the reference plane for axial imaging in everyday scanning. The creation of a standard image plane makes it easier to perform comparisons between longitudinal scans of each participant. ADNI refers to this step as standardized step but for simplification the term re-grided is used in this study. By co-registering the original frames to a standardized space in a single step, the interpolation of the image data reduces, and therefore resolution degradation caused by interpolation is minimized and is identical for all scans. It should be noted that in this step images are only spatially re-oriented and no linear scaling of the brain dimensions or non-linear wrapping is performed on the images.

**FDG PET.** Each coregistered-averaged image was reoriented into 160×160×96 grid with voxel size of 1.5 mm^3^. This orientation is based on the Talairach atlas [10]. The image grid was set such that the anterior-posterior axis of the subject is parallel to the AC-PC line. This step was done using *stereo* function in Neurostat. This function uses the FDG PET Talairach atlas template, with the default image size of 128×128×60 and voxel size of 2.25 mm^3^, to reorient the images. The AC-PC level in the template is set at slice 32 of the 60 slices (the grid size is 0-59 so this level is the 33rd slice). Since the reoriented images have a voxel size of 1.5 mm^3^, the AC-PC level should be set to a different slice. The difference between the voxel size of the reoriented image and the template is 1.5 (2.25/1.5=1.5) therefore, the AC-PC level was set to either the 48^th^ (32×1.5) or 56^th^ slice, where an 8-voxel offset is used to make sure the top of head doesn’t get cut. See figure 3S. step1. This re-grided image is used as a reference image for all the follow-up FDG PET scans for that specific participant. Furthermore in step 3, all the original frames were coregistered to this reference image using Neurostat *coreg* function, with the cost functions of 3, to create the coregistered-re-grided frames. Eventually, the coregistered-re-grided-averaged FDG PET image was generated by averaging the six coregistered-re-grided FDG frames using SPM 12, *imcal* function. See figure 3S. step2.

**Figure 3S.** Third preprocessing step for FDG PET. Step1. The coregistered-averaged FDG image (coronal, sagittal and transvers views) (left) is re-gride into 160×160×96 grid with voxel size of 1.5 mm^3^ to create the reference re-grided FDG image (coronal, sagittal and transvers views) (right). Step2. Original FDG PET frames were coregistered to this reference image to create the reference re-grided FDG. Eventually, the averaged-re-grided FDG PET image was generated by averaging the six coregistered-re-grided FDG frames.

The *stereo* function in Neurostat only has an FDG PET template to re-grid the images. Therefore, in order to re-grid AV45 scans two approaches were used:

**AV45 PET (FDG-Based).** If FDG scan was available for a participant, the FDG-based reference image was used to re-grid the coregistered-averaged AV45 image (set 2). Using Neurostat *coreg* function, the set 2 image was re-grided using cost functions of 5 or 6 (inter-modality coregistration functions). Default was cost function 6, but if there was a registration issue, cost function 5 (both functions use mutual information as a metric) was used. The AC-PC level was set to either 48 or 45. This re-grided standardized image is used as a re-grided reference image for the follow-up AV45 PET scans for that specific participant. See Figure 4S. Furthermore, all the original frames were coregistered to this reference image using the Neurostat *coreg* function, with the cost functions of 3, to create the coregistered-re-grided frames. Eventually, the coregistered-re-grided-averaged AV45 PET image was generated by averaging the four coregistered-re-grided AV45 frames using SPM 12, *imcal* function.

**AV45 PET (MRI-Based).** If FDG scan was not available for a participant, the T1-W MPRAGE MRI scan was used. SPM 12 *segmen*t function was used to segment the MRI scan into gray matter (GM) and white matter (WM). Bias regularization was set to light regularization (0.001), and Bias FWHM was set to 60 mm cutoff. Then, a weighted average image (2×GM+1×WM) was generated which resembles a skull stripped negative amyloid scan and next, it was smoothed to 8 mm PET resolution. See Figure 5S. Next this image was re-grided using Neurostat *stereo* function and served as MRI-based reference image.

Afterward, the coregistered-averaged AV45 image was coregistered to the MRI-based reference image. Using the Neurostat *coreg* function, this MRI-based reference image, successfully re-grid both amyloid negative and positive images. See Figure 6S. Furthermore, all the original frames were coregistered to this reference image using Neurostat *coreg* function, with the cost functions of 3, to create the coregistered-re-grided frames. Eventually, the re-grided-averaged AV45 PET image was generated by averaging the four coregistered-re-grided AV45 frames using SPM 12, *imcal* function.

**Figure 4S.** Third preprocessing step for AV45 PET, FDG-based. Step1. The coregistered-averaged AV45 image (coronal, sagittal and transvers views) (left) is coregistered the reference re-grided FDG image 160×160×96, voxel size of 1.5 mm^3^) to create the reference re-grided AV45 image (coronal, sagittal and transvers views) (right).

In the third preprocessing step of the ADNI pipelines, an intensity normalization for both re-grided-averaged FDG and AV45 images was implemented. FDG scans were subjected to a normalization procedure aimed at setting the global mean of a masked image to a value of 1.0. Initially, the entire image was rescaled to achieve this mean in the first iteration. In subsequent iterations, voxel values below 0.5 were masked out, and the remaining voxels were rescaled to maintain an average of 1.0. This iterative process continued until the number of masked voxels remains constant.


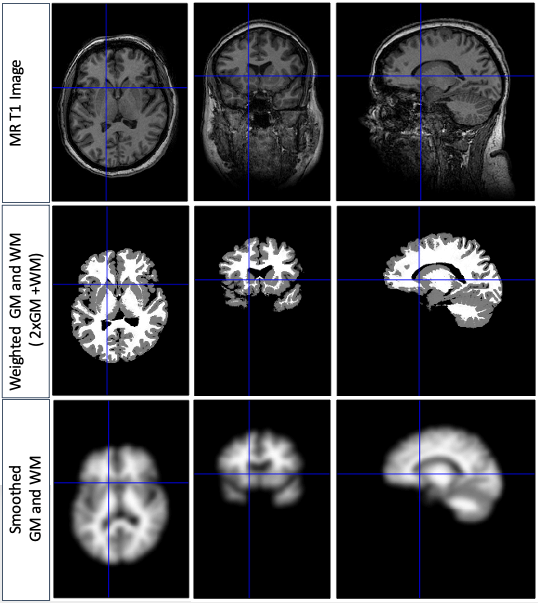


**Figure 5S.** The process of creating MRI reference image used to re-grid AV45 coregistered-averaged scans. First raw shows transverse, coronal, and sagittal views of the original MR T1-W scan, the middle row is the segmented WM and GM scan with different weights (2×GM+ 1×WM) in 3 views. The bottom row is the weighted GM and WM scan that is smoothed with an 8×8×8 mm^3^ filter in 3 views. GM; gray matter, WM; White matter.

On the other hand, AV45 images were normalized using a reference region in the cerebellar gray matter as defined by an atlas. The atlas defined reference region is applied following using the stereotactic non-linear warping routine in the NeuroStat. ADNI pipeline has a parallel in-house software that uses this warped space to define the region and get the cerebellar scale factor. The cerebellar scale factor is then applied to the amyloid re-grided-averaged image in order to normalize it. Due to use of an in-house software normalizing the amyloid images with the ADNI approach was not possible and because the SUVRs metrics were for our validation analysis, the intensity normalization was not performed.

Step 4: Coregistered, Re-grided, Averaged and Smoothed

In this step, set 3 images were smoothed so that all the images have the rough resolution of the lowest resolution scanner used in ADNI study. Consequently, scanners with higher resolution are smoothed more than the images acquired from lower resolution scanners. ADNI has measured and documented the filters for all the scanners used in their studies. Scanner-specific smoothing filter was applied to the set 3 images. The reason behind this step is to be able to compare scans across different scanners and centers.

Briefly, to achieve a uniform resolution, 3D Hoffman brain phantom was scanned with each scanner and the results were compared to a digital version of the phantom that was smoothed with an 8 mm 3D-Gaussian filter. To achieve same resolution as the digital phantom each scan was smoothed with different combination of in-plane (x-y axis) and axial (z axis) kernels. Filter of choice for each scanner was the one that yields to the highest global correlation and lowest root mean square error (RMSE). Selected filters used to smooth all the scans for each scanner model are documented and available for download from LONI website. For GE Discovery STE PET/CT, the smoothing pair filter is [5.5, 5.5] mm^2^ for in-plane and 5 mm for axial axis. This pair was used to smooth the preprocessed image set 3 for all scans, using *smooth* function in SPM 12, resulting in preprocessed set 4. See Figure 7S.

**Figure 6S.** Third preprocessing step for AV45 PET, MRI-based. Step1a. The smoothed MRI image (coronal, sagittal and transvers views) (left) is re-gride into 160×160×96 grid with voxel size of 1.5 mm^3^ to create the reference re-grided MRI image (coronal, sagittal and transvers views) (right). Step 1b. The coregistered-averaged AV45 image (coronal, sagittal and transvers views) (left) is coregistered to the reference re-grided MRI image (160×160×96, voxel size of 1.5 mm^3^) to create the reference re-grided AV45 image (coronal, sagittal and transvers views) (right).

**Figure 7S.** Re-grided-averaged image (left) was smoothed using a non-isotropic gaussian filter (right).

Region-of-Interest-Based Analyses Pipeline

Spatial normalization

**FDG PET.** Fully preprocessed FDG images were spatially normalized into MNI standard stereotactic brain atlas space using SPM12 *old normalize Estimate & write* function using its default 18F-FDG-PET template. This template is an aging and dementia-specific template that was created by nonlinear registration of a total of 120 18F-FDG PET images from 60 healthy controls (HCs) (age=69.8 ±7.49 years) and 60 patients with dementia (age=72.69±9.08 years) (Della Rosa et al., 2014). The following parameters were used in SPM: bounding box (-90, -126, -72; 91, 91, 109); voxel size (2,2,2); interpolation, Trilinear; nonlinear regularization,10. Figure 8S shows the spatially normalized image of a fully pre-procced FDG scan.

**AV45 PET.** Fully preprocessed Amyloid images were spatially normalized to MNI standard stereotactic brain atlas space with SPM12 *normalize Estimate & write* function using a specialized 18F-Florbetapir-PET template. For a detailed description of template creation see (Joshi et al., 2015). Briefly, 18F-Florbetapir-PET data from 26 subjects, 11 clinically diagnosed with AD (age range, 55–88 year) and 15 old HCs (age range, 58–86 year) were used to create the template. The following parameters were used in SPM: bounding box (-90, -126, -72; 91, 91, 109); voxel size (2,2,2); interpolation, 4th Degree B-spline; nonlinear regularization,10. See Figure 9S.


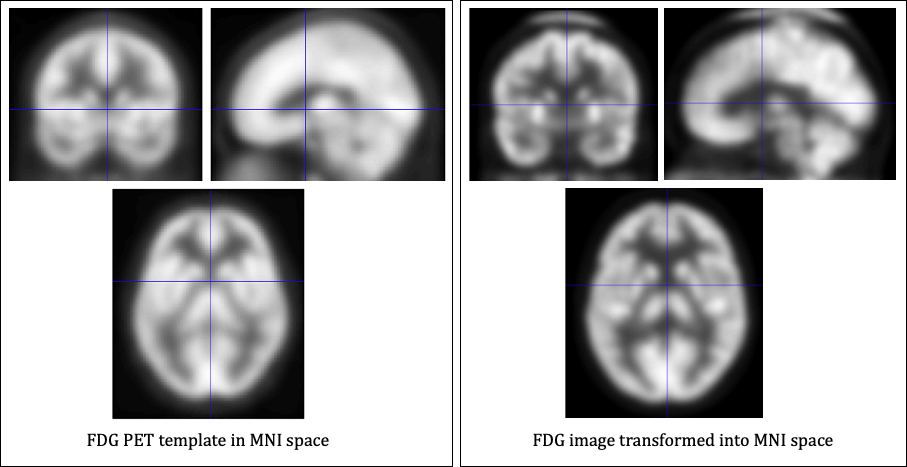


**Figure 8S.** Coronal, sagittal, and transverse views of FDG fully pre-processed image wrapped into MNI space (right).


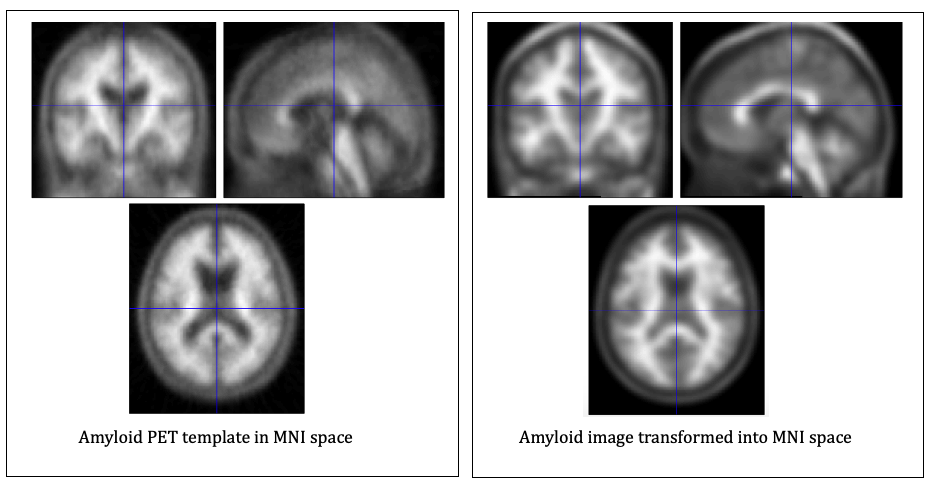


**Figure 9S.** Coronal, sagittal, and transverse views of the Amyloid PET template in MNI space (left). Coronal, sagittal, and transverse views of Amyloid fully pre-processed image wrapped into MNI space (right).

Regions of interest (ROI)

**FDG PET. The** Berkeley FDG MetaROI is a set of pre-defined ROIs created based on cited regions in literature review comparing AD, MCI and normal subjects.

**Figure 10S.** The FDG MetaROI containing 5 regions of Left Angular Gyrus, Right Angular Gyrus, Bilateral Posterior Cingular, Left Inferior Temporal Gyrus, Right Inferior, and Temporal Gyrus. Pons/Cerebellar Vermis was used as the reference region.

For the detailed description of the Meta-ROI see (Landau et al., 2011). The MetaROI containing 5 regions of Left Angular Gyrus, Right Angular Gyrus, Bilateral Posterior Cingular, Left Inferior Temporal Gyrus, Right Inferior, and Temporal Gyrus were binarized (see Figure 10S). The reference region is a hand-drawn pons/cerebellar vermis region delineated on a T1 template in MNI space. When applied on each PET scan the average of 50% of voxels with highest intensity values within the reference region was used as the reference region.

**AV45 PET.** The Avid MetaROI comprises of the Medial Orbital Frontal, Temporal, Parietal, Anterior Cingulate, Posterior Cingulate, and Precuneus. The entire cerebellum was used as the reference region. Figure 11S shows the AV45 MetaROI.

SUVR Calculation

For FDG scans, SUVRs were calculated for the 5 individual predefined ROIs as a ratio of mean uptake in the ROI with respect to the mean uptake in the pons/cerebellar vermis as the reference region. For amyloid scans SUVRs were calculated for the 6 individual predefined ROIs as a ratio of mean uptake in the ROI with respect to the mean uptake in the whole cerebellum as the reference region. The Global SUVR is simply the average of regional SUVRs referenced to the entire cerebellum. Since elevated amyloid uptake in gray matter cortical regions is a direct indication of AD, a cut off value can determine amyloid positivity or negativity. This cut off value is determined based on the goal of the study or analysis. For this study, the pre-specified cut-point of 1.10 is used based on study of Joshi et al. (Joshi et al., 2015) for cross sectional florbetapir analysis. This value is established using the whole cerebellum as the reference region and yield to an excellent classification. All the NCs with clinical diagnosis or negative pathology had a SUVR value below 1.10 and were successfully classified as AB negative. And for AB positive cases, 97% of those with pathological assessment had an SUVR value above 1.10.

**Figure 11S.** The AV45 MetaROI containing 6 regions including Medial Orbital Frontal, Temporal, Parietal, Anterior Cingulate, Posterior Cingulate, and Precuneus. Whole Cerebellum was used as the reference region.

Table 1S. Participant IDs and FDG scans information

| Participant | Exam Date | Diagnosis | In-house Global SUVR | Gold Standard Global SUVR |
| --- | --- | --- | --- | --- |
| 007-S-0101 | 3/15/2012 | MCI | 0.969 | 0.967 |
| 007-S-0698 | 8/30/2010 | MCI | 1.127 | 1.131 |
| 021-S-0159 | 4/28/2011 | NL | 1.395 | 1.395 |
| 073-S-0089 | 10/15/2010 | NL | 1.397 | 1.434 |
| 098-S-0172 | 3/16/2011 | NL | 1.291 | 1.292 |
| 098-S-0269 | 6/23/2011 | MCI | 0.749 | 0.748 |
| 098-S-0667 | 9/22/2010 | MCI | 0.787 | 0.786 |
| 100-S-0035 | 12/14/2010 | NL | 1.315 | 1.319 |
| 100-S-0069 | 1/26/2011 | NL | 1.318 | 1.32 |
| 100-S-0296 | 5/18/2011 | MCI | 1.282 | 1.281 |
| 126-S-0680 | 8/5/2011 | NL | 1.268 | 1.266 |

Table 2S. Participant IDs and AV45 scans information

| Participant | Exam Date | Diagnosis | In-house  Global SUVR | Gold Standard  Global SUVR |
| --- | --- | --- | --- | --- |
| 007-S-0101 | 2/16/2012 | MCI | 1.528 | 1.558 |
| 007-S-0698 | 8/31/2010 | MCI | 1.705 | 1.767 |
| 021-S-0159 | 4/22/2011 | NL | 1.117 | 1.125 |
| 073-S-0089 | 10/25/2010 | NL | 0.924 | 0.942 |
| 098-S-0172 | 3/22/2011 | NL | 0.892 | 0.891 |
| 098-S-0269 | 4/12/2011 | MCI | 1.303 | 1.321 |
| 098-S-0667 | 9/23/2010 | MCI | 1.310 | 1.331 |
| 100-S-0035 | 12/8/2010 | NL | 1.454 | 1.473 |
| 100-S-0069 | 1/18/2011 | NL | 0.989 | 1.004 |
| 100-S-0296 | 5/5/2011 | MCI | 1.261 | 1.272 |
| 126-S-0680 | 8/16/2011 | NL | 0.887 | 0.897 |

**References**

CHINCARINI, A., PEIRA, E., COROSU, M., MORBELLI, S., BAUCKNEHT, M., CAPITANIO, S., PARDINI, M., ARNALDI, D., VELLANI, C. & D’AMBROSIO, D. 2020. A kinetics-based approach to amyloid PET semi-quantification. *European journal of nuclear medicine and molecular imaging,* 47**,** 2175-2185.

DELLA ROSA, P. A., CERAMI, C., GALLIVANONE, F., PRESTIA, A., CAROLI, A., CASTIGLIONI, I., GILARDI, M. C., FRISONI, G., FRISTON, K. & ASHBURNER, J. 2014. A standardized [18 F]-FDG-PET template for spatial normalization in statistical parametric mapping of dementia. *Neuroinformatics,* 12**,** 575-593.

JOSHI, A. D., PONTECORVO, M. J., LU, M., SKOVRONSKY, D. M., MINTUN, M. A. & DEVOUS, M. D. 2015. A semiautomated method for quantification of F 18 florbetapir PET images. *Journal of Nuclear Medicine,* 56**,** 1736-1741.

LANDAU, S. M., HARVEY, D., MADISON, C. M., KOEPPE, R. A., REIMAN, E. M., FOSTER, N. L., WEINER, M. W., JAGUST, W. J. & INITIATIVE, A. S. D. N. 2011. Associations between cognitive, functional, and FDG-PET measures of decline in AD and MCI. *Neurobiology of aging,* 32**,** 1207-1218.
